# Apathy and effort‐based decision‐making in Alzheimer's disease and subjective cognitive impairment

**DOI:** 10.1002/dad2.70013

**Published:** 2024-10-16

**Authors:** Bahaaeddin Attaallah, Sofia Toniolo, Maria Raquel Maio, Masud Husain

**Affiliations:** ^1^ Nuffield Department of Clinical Neurosciences University of Oxford Oxford UK; ^2^ Centre for Preventive Neurology Queen Mary University of London London UK; ^3^ Department of Experimental Psychology University of Oxford Oxford UK

**Keywords:** Alzheimer's disease, apathy, drift diffusion modeling, effort‐based decision making, resting‐state fMRI, subjective cognitive impairment

## Abstract

**INTRODUCTION:**

Apathy is a significant feature in Alzheimer's disease (AD) and subjective cognitive impairment (SCI), though its mechanisms are not well established.

**METHODS:**

An effort‐based decision‐making (EBDM) framework was applied to investigate apathy in 30 AD patients, 41 SCI participants, and 55 healthy controls (HC). Data were analyzed using a drift‐diffusion model (DDM) to uncover latent psychological processes.

**RESULTS:**

SCI participants reported higher apathy than AD patients and HC. However, informant reports of apathy in AD patients were higher than self‐reports and indicated significant apathy compared to HC. Both the AD and SCI groups showed reduced sensitivity to effort changes, linked to executive dysfunction in AD and apathy in SCI. Increased resting functional cortical connectivity with the nucleus accumbens (NA) was associated with higher apathy in SCI.

**DISCUSSION:**

These results highlight a similar disruption of EBDM in AD and SCI, differentially related to executive functioning in AD and apathy in SCI.

**Highlights:**

- This is the first study investigating apathy using an effort‐based decision‐making (EBDM) framework in Alzheimer's disease (AD) and subjective cognitive impairment (SCI).
- Self‐reports underestimate apathy in AD patients when compared to informant reports and healthy controls (HC). SCI participants, in whom self and informant reports were more concordant, also showed higher degrees of apathy.
- Both AD and SCI groups showed reduced sensitivity to effort.
- Reduced sensitivity to effort correlates with executive dysfunction in AD and apathy, but not depression, in SCI.
- Increased nucleus accumbens (ventral striatum) connectivity with the frontoparietal network was associated with higher apathy scores in SCI.
- The results thus suggest that while AD and SCI can have similar deficits in EBDM, these deficits correlate with distinct clinical manifestations: executive dysfunction in AD and apathy in SCI.

## 1 INTRODUCTION

Apathy (ie, diminished goal‐directed behavior) is a prominent feature in several neurodegenerative disorders, including Alzheimer's disease (AD), Parkinson's disease (PD), and small vessel disease (SVD).^1^ It burdens patients and caregivers and is a marker of disease progression in some disorders.^2^, ^3^

In AD, up to 80% of patients develop apathy, even at early stages, making it a valuable preclinical marker.^4^ However, it remains poorly understood in AD, with limited behavioral studies and no licensed treatments despite some positive clinical trials.^5^, ^6^

Subjective cognitive impairment (SCI) is often linked to affective burden (eg, depression) despite significant evidence suggesting differences between the two syndromes.^7^ This distinction is crucial, as apathy, particularly without depression, increases the risk of progression from MCI to AD^8^ and has been independently linked to SCI to MCI progression.^7^ This differential impact of apathy on dementia conversion necessitates mechanistic characterization.

The syndrome of apathy can occur in several different domains: behavioral, cognitive, emotional, and social.^9^, ^10^ The best studied of these is behavioral apathy which includes deficits in processing of rewards and costs, driving toward goals.^11^ Studies suggest that it may arise from hyposensitivity to rewards and/or hypersensitivity to costs, such as effort.^12^ These effects can potentially reduce goal‐directed behavior by making more actions seem “less worthy.” The effort‐based decision‐making (EBDM) framework and its modeling have been pivotal in advancing our understanding of motivation in neurological conditions.^11^, ^12^, ^13^ For instance, in PD, EBDM studies have identified decreased incentivization by reward, as opposed to increased effort aversion, as a key factor in apathy, but not in dysphoria associated with depression.^14^ Similar methodologies have been applied to other conditions such as SVD, where again the effects of reward and effort on motivational drive correlated with apathy, but not depression.^15^, ^16^

Cognitive apathy, on the other hand, has been considered to result from deficits in utilizing cognitive resources to plan decisions and actions toward goals.^9^, ^10^, ^11^ This could stem from a range of executive and attentional deficits, leading to dysfunctional cognitive effort allocation and ultimately biasing cost/benefit sensitivity in EBDM.^17^, ^18^ These deficits can be observed in patients with cognitive impairment and dementia,^19^, ^20^, ^21^ and are possibly related to different brain neuroanatomical and neurochemical systems compared to behavioral apathy.^9^ While dopaminergic and serotonergic systems and associated mesocorticolimbic regions have been linked to motivational drive for behavioral goals,^22^, ^23^ cholinergic and noradrenergic pathways featuring brain regions involved in executive functioning might be significant in the development of cognitive apathy.^24^, ^25^

Apathy in AD is linked to various brain regions that support these motivational or cognitive aspects of goal‐directed behavior.^26^, ^27^ Structurally, this involves atrophy and microstructural disruptions of cortical and subcortical regions (eg, frontal, anterior cingulate, limbic, and striatal regions) or their connecting tracts.^28^, ^29^ The disruption of these areas is often accompanied by functional connectivity deficits that have been consistently implicated in apathy, such as fronto‐striatal and salience networks.^26^, ^30^, ^31^ These different neuroimaging features have been linked to EBDM in other conditions.

To our knowledge, the EBDM approach has not been used to investigate apathy in AD or SCI. This study aims to examine EBDM in these groups and its association with apathy and brain functional connectivity.

## 2 METHODS

### 2.1 Participants

The study included 30 patients with a clinical diagnosis of AD (14 females), 41 participants with SCI (19 females), and 55 healthy controls (HC) (30 females).

Informed consent was signed by all participants and monetary compensation was offered for participation. The study was approved by the Ethics Committee at the University of Oxford (RAS ID: 248379, Approval Reference: 18/SC/0448).

### 2.2 Clinical measures and procedures

All participants were cognitively evaluated using Addenbrooke's Cognitive Examination‐III (ACE‐III) and digit span (DS). The DS requires participants to repeat a series of numbers of increasing length. The forward version assesses simple attention by asking participants to repeat the numbers in the order in which they hear them. The backward (reverse) version is thought of as an index of executive function, as participants recruit working memory resources to mentally manipulate the numbers and repeat them in *reverse* order,^32^ in addition to potential attentional processes generally involved in both versions. Participants also completed self‐report measures of apathy (Apathy Motivation Index [AMI])^33^ and depression (Beck's Depression Inventory [BDI‐II]).^34^ The AMI is an 18‐item questionnaire that has been used previously in healthy people and various neurological conditions to measure apathy and its dimensions including behavioral, social, and emotional.^35^, ^36^ Reports on SCI and AD patients’ apathy were also obtained from informants when available using the AMI caregiver version (AMI‐CG).^35^

**RESEARCH IN CONTEXT**

1. **Systematic review**: Online databases (PubMed and Google Scholar) were used to search for articles investigating apathy in Alzheimer's disease (AD) and subjective cognitive impairment (SCI). Our study is the first to utilize effort‐based decision‐making (EBDM) to study the syndrome in these two groups.
2. **Interpretation**: Our findings highlight a similar deficit in EBDM across AD and SCI that relates to apathy and executive dysfunction.
3. **Future directions**: Future studies should longitudinally investigate EBDM deficits to determine their prognostic value for the risk of progression to AD. Other studies might investigate alternative mechanisms of apathy using similar behavioral paradigms and approaches.

### 2.3 Experimental paradigm and procedure

**FIGURE 1:**
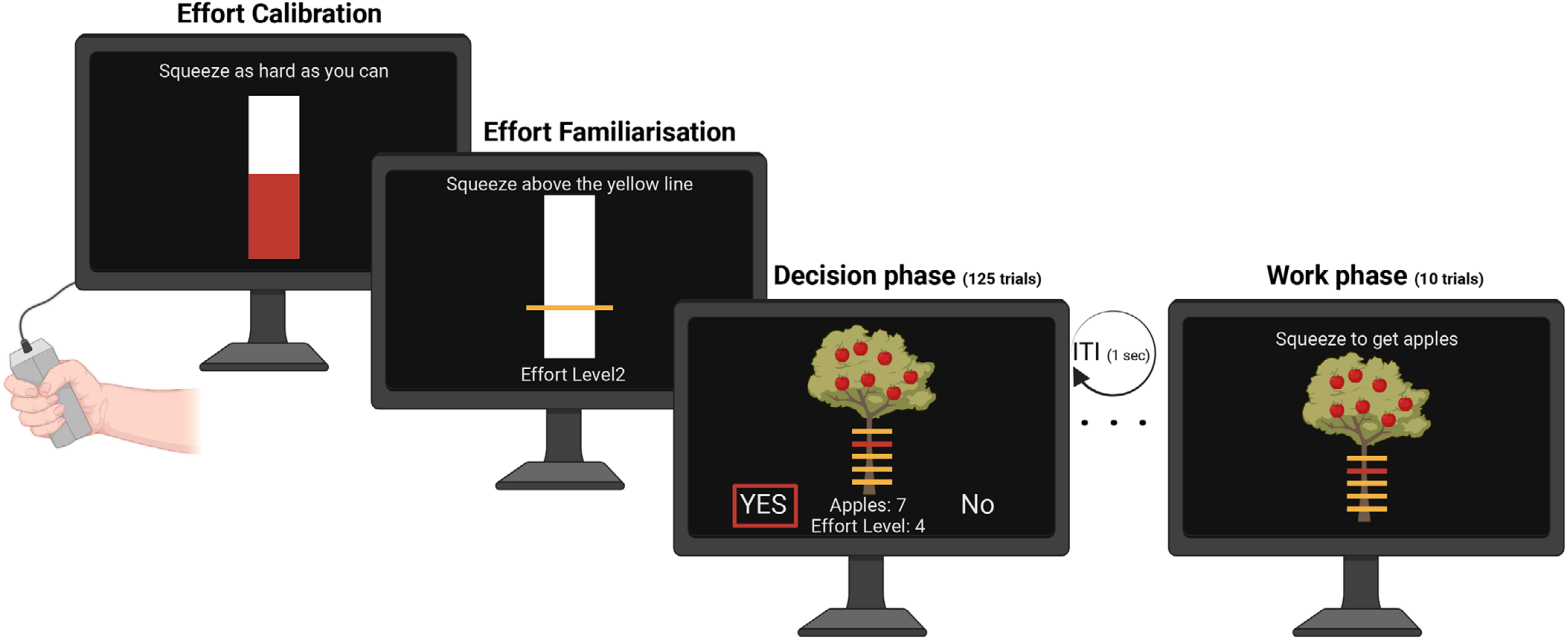
EBDM task. First, MVC was obtained to be used for effort level calibration for each participant. Effort familiarization involved practicing the different effort levels twice per level. There were five effort levels indicated by yellow lines on a tree (or bar) in ascending order. During the decision‐making phase, participants responded to offers (yes or no) using arrow keys to indicate whether the reward (apples) on offer was worth the effort. In the work phase, 10 random offers were selected from the participants’ responses to play and obtain apples. EBDM, effort‐based decision‐making; MVC, maximum voluntary contraction.

A short version of an extensively used and validated EBDM paradigm was used (Figure 1).^14^, ^16^ The task examines how people respond to offers (accept/reject), assessing whether the reward on offer is worth the effort needed to obtain it. There were five reward levels visualized as virtual apples on a tree (1, 4, 7, 10, and 13) and five effort levels visualized as bars on a tree trunk. The higher the bar on the tree trunk, the higher the effort level. These levels corresponded to a percentage (16%, 32%, 48%, 64%, and 80%) of participants’ maximum voluntary contraction (MVC) that was measured and recorded at the start of the experiment using a handheld dynamometer. There were 25 conditions of the different reward and effort combinations (5 × 5), each repeated five times in five blocks giving rise to 125 trials. Trials were randomized across these blocks and according to the position of the “Yes” and “No” on the screen. See Supplementary Methods for further details.

### 2.4 Statistical and behavioral analyses

The initial analyses of raw behavioral data (choices and decision times [DTs]) were performed using mixed‐effect models fitted using *fitglm* in MATLAB v2021a, controlling for age and gender. A quadratic expression of effort was used similar to previous comparable studies.^37^ DTs were log‐transformed to achieve normality and trimmed with the exclusion of outliers that were three standard deviations above or below the group mean.

Robust linear regression models using *fitlm* functions in MATLAB were used to investigate the correlations within groups. For exploratory analyses, family‐wise error rate correction was performed for six multiple comparisons within each group using the Bonferroni method.

All frequentist statistical tests were two‐tailed with a testing level (alpha) of 0.05.

Further statistical details and description of models and their results are reported in the Supplementary Information (TablesS1,S2).

#### 2.4.1 Drift diffusion modeling

Combining choices and DTs provides a comprehensive understanding of behavioral differences and latent psychological processes in decision‐making. This was achieved using the hierarchical drift diffusion model (DDM), which has been validated in studies on SVD^16^ and schizophrenia.^38^ The model parameterizes decision‐making as a noisy accumulation of evidence toward acceptance or rejection.

The evidence accumulation rate (drift rate, *v*) has a linear relationship with reward (*r*) and squared effort (*e*):

(1) $$v_{r,e}=v_{0}+v_{r}-v_{e}-v_{r*e}$$

The model returned seven parameters (Table S3): the influence of reward (*v_r_*), effort (*v_e_*), and their interaction (*v_r×e_*) on drift rate; basic drift rate (*v*_0_); decision threshold (*a*); initial bias (*z*); and nondecision time (*t*). Parameters *a*, *z*, and *t* were constant across conditions.

Data fitting was performed with the HDDM toolbox (http://ski.clps.brown.edu/ hddm_docs/; version 0.6.0; with Python 3). Bayesian significance testing examined posterior probabilities, with *>*0.95 considered significant.

### 2.5 Magnetic resonance data acquisition

A 3T Siemens Verio scanner was used to obtain structural and functional magnetic resonance scans at John Radcliff Hospital, Oxford. Structural images were 1‐mm isotropic T1‐weighted (MPRAGE, field of view: 208 × 256 × 256 matrix, TR/TE = 200/1.94 ms, lip angle = 8°, iPAT = 2, prescan‐normalize). Resting‐state functional magnetic resonance imaging (rs‐fMRI) measures spontaneous changes in blood oxygenation (BOLD signal) due to intrinsic brain activity. The rs‐fMRI images were a voxel size of 2.4 × 2.4 × 2.4 mm (GE‐EPI with multiband acceleration factor = 8, field of view: 88 × 88 ×  64 matrix, TR/TE = 735/39 ms, flip angle = 52°, fat saturation, no iPAT).

MR images were obtained from all MRI‐compatible participants who consented to be scanned for the study. This included 43 HC, 24 AD patients, and 30 SCI participants.

### 2.6 Magnetic resonance data analyses

The CONN toolbox v20.b^39^ running SPM12 was used for the analysis of resting‐state connectivity analysis in MATLAB 2021a, with the implementation of the default processing pipeline (see details in the Supplementary Information).

A region of interest (ROI)–ROI functional connectivity analysis was performed. The ROIs were 26 ROIs representing key nodes of the main known cortical functional networks, including salience, default mode, sensorimotor, dorsal attention, and frontoparietal networks (FPN), in addition to subcortical regions of relevance such as the hippocampus (a key pathological region in AD) and the nucleus accumbens (NA) (ventral striatum) regions (a key subcortical region involved in EBDM). These regions and their names were defined based on default masks in the CONN toolbox. Overall, 325 connections were examined, controlling for age and gender differences. Significance testing was performed using threshold‐free cluster enhancement (TFCE) with a corrected cluster significance threshold equal to 0.05.

## 3 RESULTS

### 3.1 Demographics and apathy scores

**FIGURE 2:**
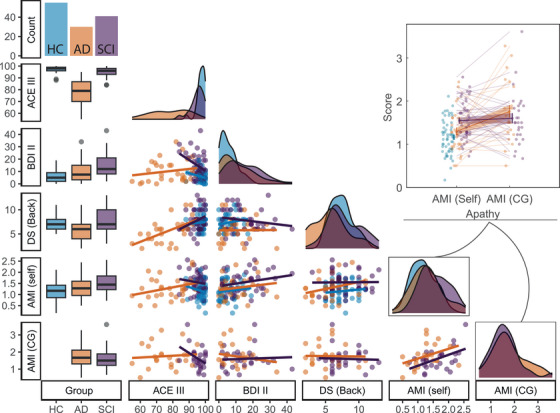
Demographics. AD patients had lower ACE‐III and DS spans than the other two groups. While AD patients were not different in their AMI and BDI‐II scores from HC, SCI participants were significantly more apathetic than HC and AD, and more depressed than HC. Apathy scores obtained from AD patients’ caregivers were higher than subjective scores. ACE‐III, Addenbrooke's Cognitive Examination III; AD, Alzheimer's disease; AMI, Apathy Motivation Index; AMI (CG), AMI caregiver version; BDI‐II, Beck's Depression Inventory‐II; DS, digit span; HC, healthy controls.

Study demographics are summarized in Table 1 and Figure 2.

**TABLE 1:** Study demographics.

	HC 55 (25/30)		AD 30 (16/14)		SCI 41 (22/19)			Post hoc *p*‐value NA		
N (M/F)	Mean	SD	Mean	SD	Mean	SD	*p*‐value 0.67	HC vs AD	HC vs SCI	AD vs SCI
Age (Y)	62.11	10.04	68.67	9.91	58.17	7.82	*<*0.001	0.03	0.14	*<*0.001
Education (Y)	16.98	5.60	13.93	4.08	15.93	4.50	0.02	0.02	0.79	0.16
ACE‐III	97.35	2.41	78.20	11.18	95.00	3.94	*<*0.001	*<*0.001	0.02	*<*0.001
DS	17.81	2.72	15.47	4.28	18.83	4.16	*<*0.01	0.01	0.91	*<*0.01
DS forward	10.45	1.91	9.70	2.23	11.00	2.28	0.04	0.29	0.44	0.03
DS backward	7.36	1.63	5.77	2.50	7.83	2.39	*<*0.01	*<*0.01	0.91	*<*0.01
AMI	1.16	0.40	1.30	0.49	1.55	0.46	*<*0.001	0.36	*<*0.001	0.049
BDI‐II	5.75	4.72	10.30	8.82	14.88	9.78	*<*0.001	0.07	*<*0.001	0.07
AMI‐CG^a^	–	–	1.76	0.70	1.60	0.62	0.44	NA		

Abbreviations: ACE‐III, Addenbrooke's Cognitive Examination‐III; AD, Alzheimer's disease; AMI, Apathy Motivation Index; AMI‐CG, AMI caregiver version; BDI‐II, Beck's Depression Inventory‐II; DS, digit span; HC, healthy controls; M/F, male/female; SCI, subjective cognitive impairment; Y, years.

^a^ Collected for 23 AD patients and 27 SCI participants.

There was no significant difference between AD and HC in self‐reported apathy (AMI) or depression (BDI‐II) (AMI; HC: *μ *= 1.16, standard deviation [SD] = 0.40, AD: *μ *= 1.30, SD = 0.49, SCI: *μ *= 1.55, SD = 0.46, *p < *0.001 for HC vs SCI, *p *= 0.049 for AD vs SCI; BDI‐II; HC: *μ *= 5.75, SD = 4.72, AD: *μ *= 10.30, SD = 8.82, SCI: *μ *= 14.88, SD = 9.78, *p < *0.001 for HC vs SCI, *p *= 0.07 for AD vs SCI). By contrast, SCI participants reported significantly higher levels of apathy (AMI) compared to the other two groups, and higher depression compared to HC.

Because the AD disease spectrum might be associated with decreased insight,^40^ apathy reports were also collected from informants for AD and SCI groups (AD: *N *= 23, SCI: *N *= 27) using the recently developed AMI‐CG.^35^ These informant reports showed that AD patients were rated as being significantly more apathetic than indicated by self‐reports (AMI‐CG: *μ *= 1.76, SD = 0.70, AMI: 1.30, SD = 0.49, *t*(22) = 3.59*, p *= 0.0016; Figure 2). These informant scores in AD were also generally higher than AMI scores in HC, revealing that AD patients are more apathetic than HC when considering external apathy reports (*t*(76) = 4.75*, p < *0.001). There was no significant difference between AD and SCI AMI‐CG reports (*p *= 0.44). Similarly, SCI self‐reported apathy scores were not significantly different from informant reports (*p *= 0.85). The two AMI versions were correlated within the two groups (Robust regression *AMI ∼ AMI‐CG*; AD: *β *= 0.28*, t*(21) = 2.35*, p *= 0.028*, R* ^2^ = 0.22, SCI:*β *= 0.39*, t*(25) = 2.73*, p *= 0.011*, R* ^2^ = 0.231). There was no significant correlation between apathy (self‐ or informant‐reported) and depression in any of the groups.

### 3.2 Lower sensitivity to effort in AD and SCI

**FIGURE 3:**
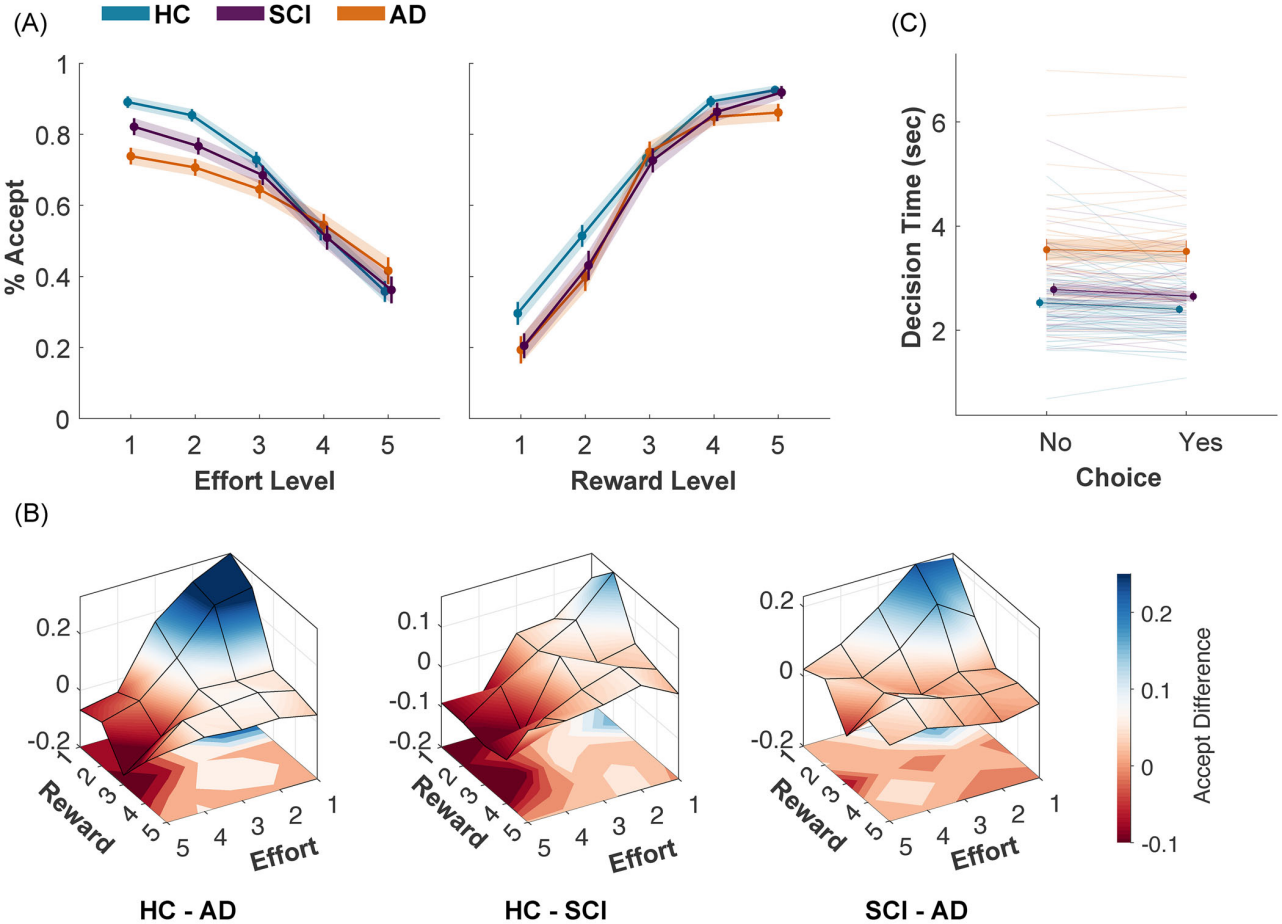
Differences in acceptance and decision time between AD, SCI, and HC. (A) Participants from the three groups responded to offers according to their effort and reward attributes, accepting more when effort decreased and reward increased. Differential acceptance slopes as a function of effort are seen across the three groups, with AD patients showing reduced sensitivity to effort (shallower slopes) compared to HC and SCI. (B) These differences resulted in different acceptance maps across the decision space, that is, AD patients showed reduced acceptance of low‐reward, low‐effort offers compared to HC (blue region in the left‐hand heatmap). (C) AD patients and SCI participants were generally slower than HC in making decisions; this was the case for both accept and reject decisions. Error bars show SEM ± SD. See Tables S1,S2 for statistical details. AD, Alzheimer's disease; HC, healthy controls; SCI, subjective cognitive impairment.

Participants modulated their responses to reward and effort: accepting more offers with higher rewards and lower efforts (Main effect of reward: *β *= 3.04, 95% confidence interval [CI] = [2.64, 3.43]*, t*_15721_ = 15.08*, p < *0.0001; Main effect of effort: *β *= −2.34, 95%*CI *= [−2.67, ‐2.01]*, t*_15721_ = −13.91*, p < *0.0001; Figure 3A,B, Table S1).

AD participants’ acceptance of offers was less influenced by effort compared to HC and SCI (AD × Effort vs HC: *β *= +1.21, 95% CI = [0.66, 1.78]*, t*_15721_ = 4.30*, p < *0.0001; AD × Effort vs SCI: *β *= 0.80, 95% CI = [0.18, 1.41]*, t*_15721_ = 2.55*, p *= 0.01; Figure 3A,B, Tables S1,S2). AD patients were less likely to accept low‐effort offers (mean acceptance at lowest effort: 89.02%, 82.15%, and 73.51% for HC, SCI, and AD, respectively). The influence of reward was not significantly different between AD and HC (*p *= 0.058) but differed significantly between AD and SCI (AD × Reward vs SCI: *β *= −1.11, 95% CI = [−1.85, −0.36]*, t*_15721_ = −2.91*, p *= 0.003; Table S2).

SCI participants did not show significant differences compared to HC in offer acceptance or the influence of effort and reward (all *p > *0.1).

DTs were slower with increased effort (Main effect of effort: *β *= 0.04*, t*_15650_ = 6.46, 95% CI = [0.03, 0.059]*, p < *0.0001; Figure 3C, Table S1). This was more pronounced with high‐reward, high‐effort decisions (Effort × reward: *β *= 0.05*, t*_15650_ = 10.40, 95% CI = [0.04, 0.06]*, p < *0.0001; Figure 3C, Table S1). AD patients were significantly slower than HC, regardless of offer attributes (Main effect of AD: *β *= 0.28*, t*_15650_ = 5.86, 95% CI = [0.21, 0.43]*, p < *0.001; Figure 3, Table S1). SCI participants were also slower than HC (Main effect of SCI: *β *= 0.12*, t*_15650_ = 2.51, 95% CI = [0.02, 0.22]*, p *= 0.012) but faster than AD (*β *= 0.20*, t*_15650_ = 3.28, 95% CI = [0.08, 0.32]*, p *= 0.001).

### 3.3 Drift diffusion model

**FIGURE 4:**
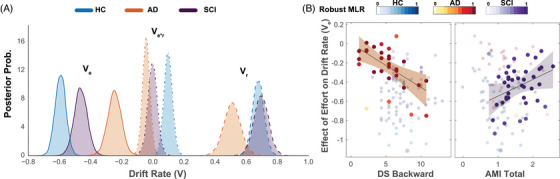
DDM results. (A) Hierarchical DDM showed that the drift rate in the study groups was influenced by both effort and reward. The effect of effort on drift rate (*V_e_*) was significantly weaker in AD patients compared to SCI and HC groups as well as in SCI compared to HC. Similarly, but to a lesser extent, the effect of reward (*V_r_*) was weaker in AD patients compared to SCI but was not different between SCI and HC. (B) A robust linear regression controlling for age, gender depression, and cognitive function showed that reduced sensitivity to effort indexed by lower *V_e_* correlated with executive dysfunction indexed by digit span (DS) backward scores, but not apathy. By contrast, reduced sensitivity to effort was significantly correlated with apathy (AMI score) in the SCI group. The color grading represents the contribution of individual data points to the robust multiple linear regression (MLR) which down‐weights multivariate outliers. AD, Alzheimer's disease; AMI, Apathy Motivation Index; DDM, drift diffusion model; HC, healthy controls; SCI, subjective cognitive impairment; *V_r_*, the effect of reward on *V*; *V_r∗e_*, the effect of interaction between reward and effort on *V*.

The DDM results indicated that effort's effect on drift rate (*v_e_*) differed significantly among the groups. AD patients showed weaker effects of effort on drift rate compared to both HC (AD vs HC: *Mean diff *= 0.34, SD = 0.056, 95% CI = [0.23, 0.45]*, P_P |D_ > *0.99, Figure 4A) and SCI (AD vs SCI: *Mean diff *= 0.21, SD = 0.061, 95% CI = [0.097, 0.33]*, P_P |D_ > *0.99). SCI also showed weaker effort effects than HC (SCI vs HC: *Mean diff *= 0.13, SD = 0.054, 95% CI = [0.024, 0.23]*, P_P |D_ > *0.99).

Reward effects on drift rate did not differ between HC and SCI (SCI vs HC: *Mean diff *= 0.019, SD = 0.059, 95% CI = [−0.096, 0.13]*, P_P |D_ ≈* 0.37), but were weaker in AD compared to both groups (AD vs HC: *Mean diff *= −0.16, SD = 0.066, 95% CI = [−0.29, −0.034]*, P_P |D_ > *0.99; AD vs SCI: *Mean diff. *= −0.186, SD = 0.073, 95% CI = [−0.32, −0.043]*, P_P |D_ > *0.99). Within the AD group, the reward effect on drift rate was more significant than the effort effect (*P_P |D_ > *0.99). These results show again that AD patients were less sensitive to changes in effort in their decision‐making process, and reveal a similar effect in SCI patients, now that both preferences and DTs are taken into consideration.

AD patients also showed differences in other model parameters compared to HC, including lower decision threshold (*a*(AD vs HC): *Mean diff. *= 0.32, SD = 0.14, 95% CI = [0.046, 0.61]*, P_P |D_ ≈* 0.99) and longer non‐DTs (*t*(AD vs HC): *Mean diff. *= 0.32*, SD *= 0.10, 95% CI = [0.125, 0.53]*, P_P |D_ > *0.99). No significant differences were found in decision bias (*z*) across groups.

### 3.4 Reduced effort sensitivity correlates with executive dysfunction in AD and with apathy in SCI

Using robust multiple linear regression, we investigated whether effort sensitivity indexed by *v_e_* correlated with self‐reported apathy (AMI), cognitive and executive function, depression, age, and gender.

In AD patients, self‐reported apathy (AMI) did not significantly correlate with any DDM parameters (all *p > *0.45). Effort sensitivity (*v_e_*) significantly correlated with executive function as indexed by DS backward score (*β *= −0.04*, t *= −2.11*, p *= 0.04; Figure 4B). No significant correlations were found with global cognition, age, gender, or depression.

In SCI participants, reduced effort sensitivity was associated with self‐reported apathy scores (*β *= 0.24*, t *= 3.01*, p *= 0.004), but not with depression, global cognition, or executive function. The correlation with apathy was primarily driven by behavioral apathy (*β *= 0.15*, t *= 2.82*, p *= 0.004).

Repeating the analysis with AMI‐CG scores instead of AMI did not reveal significant findings. Further exploratory analyses correcting for multiple comparisons investigated the relationship between other DDM parameters and apathy scores (AMI and AMI‐CG). No significant findings were observed between other parameters and apathy scores in any group.

In summary, reduced sensitivity to effort in AD correlates with deficits in executive functioning rather than self‐reported apathy, whereas in SCI, self‐reported apathy significantly relates to reduced effort sensitivity.

### 3.5 Apathy is associated with disrupted connectivity between the NA and the front‐parietal network in SCI

**FIGURE 5:**
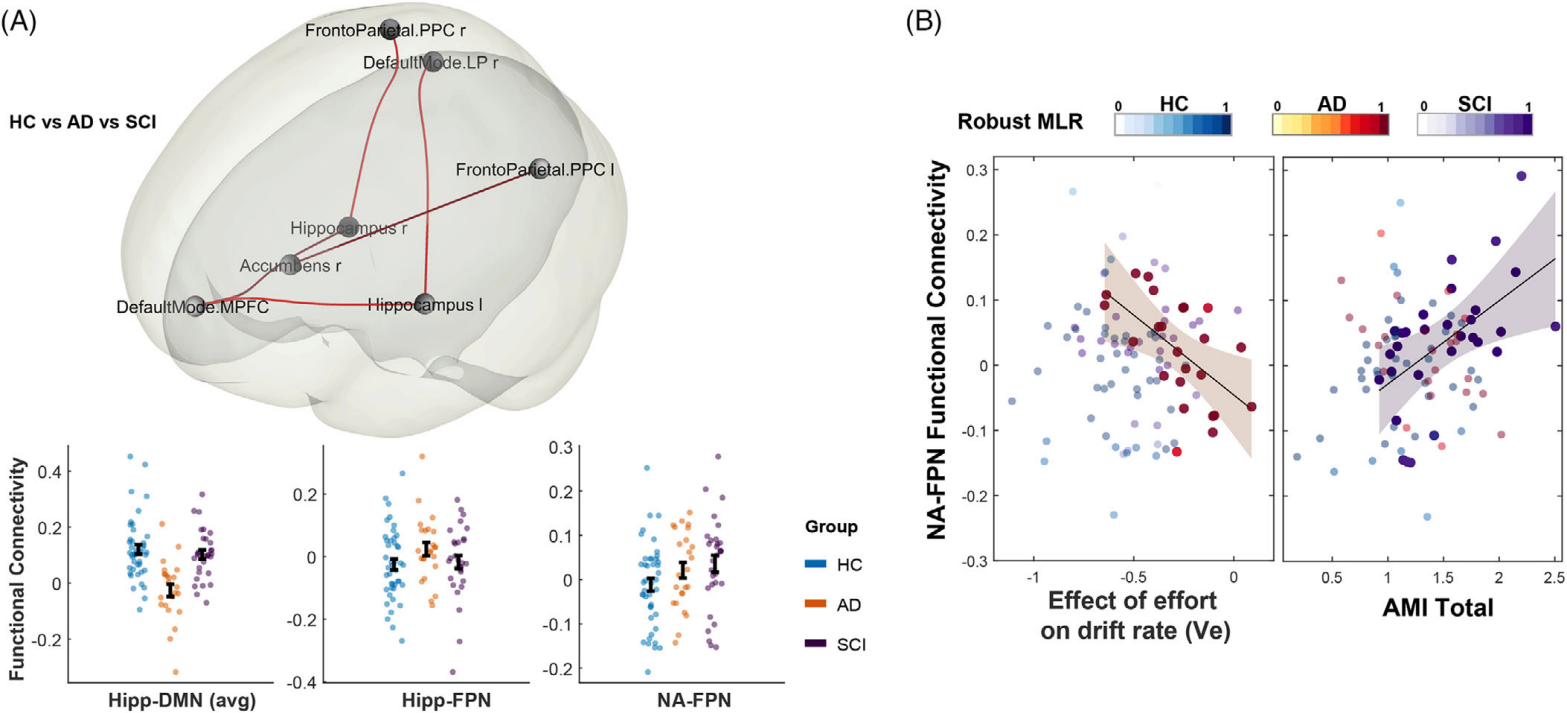
Functional connectivity differences and relationship with apathy. (A) The results showed that the three groups differ in a specific cluster that includes connections between the Hipp, NA (ventral striatum), DMN, and FPN. Compared to HC, AD had decreased Hipp‐DMN connectivity while SCI had increased NA‐FPN.PPC connectivity. (B) Increased NA‐FPN.PPC is associated with increased apathy in SCI and increased effort sensitivity in AD, indexed by *V_e_*. AD, Alzheimer's disease; AMI, Apathy Motivation Index; Hipp, hippocampus; DMN, default mode network; FPN, frontoparietal network; HC, healthy controls; NA, nucleus accumbens; SCI, subjective cognitive impairment; *V_e_*., the effect of effort on drift rate.

Functional connectivity analysis among the 26 ROIs identified one significant cluster differentiating the three groups (HC, AD, SCI: TFCE = 18.88*, p_unc_ < *0.001*, p_F WE_ *= 0.01, Figure 5A,B). This cluster highlighted five significant connections, summarized in Table 2. The first three connections involved reduced hippocampal‐DMN connectivity in AD compared to the other two groups (AD vs HC: *z *= ‐3.34*, p_unc_ < *0.001*, p_corr_ *= 0.0075; AD vs SCI: *z *= −2.86*, p_unc_ *= 0.004*, p_corr_ *= 0.037). The fourth connection involved the hippocampus and the posterior parietal cortex (PPC) within the FPN, but this difference was not statistically significant between the groups in isolation. The fifth connection involved increased connectivity between the NA and FPN.PPC in SCI compared to HC, though it did not survive multiple comparison corrections (*z *= 2.15*, p_unc_ *= 0.030*, p_corr_ > *0.05).

**TABLE 2:** Functional connectivity differences between the three groups (HC vs AD vs SCI).

Analysis Unit	Statistic	p‐unc	p‐FDR p‐FWE
Cluster 1/1	TFCE = 18.88	0.000497	0.016904 0.014000
Connection Hippocampus r ‐DefaultMode.MPFC (1,55,‐3)	F(2,92) = 12.15	0.000021	0.006758
Connection Hippocampus l ‐DefaultMode.MPFC (1,55,‐3)	F(2,92) = 7.50	0.000958	0.054623
Connection Hippocampus l ‐DefaultMode.LP (R) (47,‐67,29)	F(2,92) = 5.29	0.006690	0.135882
Connection Hippocampus r ‐FrontoParietal.PPC (R) (52,‐52,45)	F(2,92) = 2.48	0.089209	0.427206
Connection Accumbens r ‐FrontoParietal.PPC (L) (‐46,‐58,49)	F(2,92) = 2.42	0.094192	0.427206

Abbreviations: AD, Alzheimer's disease; FDR, false discovery rate; FEW, family‐wise error rate; HC, healthy controls; LP, lateral parietal; MPFC, medial prefrontal cortex; PPC, posterior parietal cortex; SCI, subjective cognitive impairment; TFCE, threshold‐free cluster enhancement; unc, uncorrected.

Using a robust regression model controlling for age, gender, depression, executive function, and global cognition, we explored the relationship between these connections and apathy (AMI scores). Increased NA‐FPN.PPC connectivity correlated with higher apathy scores in SCI (*β *= 0.12*, t*(22) = 2.8*, p_unc_ *= 0.008*, p_corr_ *= 0.048). No other significant correlations were found, including with executive function.

In AD, NA‐FPN.PPC connectivity was correlated with effort sensitivity (*β *= −0.25*, t*(22) = −2.80*, p_unc_ *= 0.011), but this did not survive correction (*p_corr_ *= 0.06).

The correlation between functional connectivity and AMI‐CG was not investigated due to insufficient correlation with task measures and a small sample size.

These findings suggest a specific network linked to apathy and EBDM dysfunction across the AD‐SCI spectrum.

## 4 DISCUSSION

Apathy is highly prevalent in AD and emerges early, even in prodromal stages such as MCI and SCI, serving as a predictor for dementia progression.^4^ In MCI, apathy is associated with a higher conversion risk to AD and greater cognitive decline, suggesting it is an important aspect of the disease process.^2^ In SCI, apathy indicates future cognitive deterioration and the likelihood of developing MCI and AD.^7^ Clinically, apathy impacts the quality of life, increases caregiver burden, and complicates disease management.^2^, ^3^

Previous investigations have linked apathy to dysfunctional EBDM in other neurodegenerative disorders, but such a characterization in AD and SCI is still lacking.^11^, ^15^, ^16^ This study used an EBDM paradigm and functional neuroimaging to investigate apathy in AD and SCI.

AD participants did not report higher apathy scores, but caregiver reports indicated significant apathy compared to HC. SCI participants reported higher levels of apathy, consistent with informant estimations. On the EBDM task, both AD and SCI participants demonstrated reduced sensitivity to effort, correlating with apathy in SCI and executive dysfunction in AD. Functional connectivity analysis revealed differences across different networks, including a connection involving the NA and PPC in the FPN, which correlated with apathy severity in SCI.

Reduced effort sensitivity in AD might indicate lower weights assigned to effort changes when making decisions, reflecting reduced motivational drives (ie, behavioral apathy). This decision‐making profile is similar to apathetic SCI individuals. However, these deficits in AD were related to executive dysfunction rather than apathy scores, suggesting an inability to compute subjective values involving multiple attributes simultaneously. This aligns with the concept of cognitive apathy.^9^, ^10^, ^23^ See additional discussion on this in the Supplementary Information.

Apathy, but not depression, in SCI, was specifically related to blunted effort sensitivity. This offers further mechanistic insights into affective and motivational functioning in the condition and aligns with a broad body of evidence emphasizing the distinction between apathy and depression.^41^ For example, we have previously reported that affective functioning in SCI might be related to increased reactivity to uncertainty and faster decisions, contrary to what apathetic SCI individuals have demonstrated in this study with slower EBDM times.^42^ The distinction between apathy and depression in this context might be critical as many reports link apathy, not depression, to the risk of progression to dementia including recent SCI longitudinal studies.^7^ Our results highlight a behavioral objective marker (reduced sensitivity to effort) of apathy in this group, which might be important for prognostication. This is potentially important also in AD patients for whom self‐reports of apathy might not be reliable. It might be crucial in the future to conduct longitudinal studies to investigate whether EBDM markers have any prognostic value on the progression to MCI and AD. Similarly, conducting cross‐sectional research involving MCI is invaluable for a better understanding of these deficits across the full AD spectrum.

The NA (ventral striatum) plays a pivotal role in EBDM by integrating motivational and reward‐related information, facilitated through its connections with the prefrontal cortex (PFC) and the limbic system.^11^, ^13^ This mesocorticolimbic integration could influence the likelihood of choosing more effortful options when potential rewards are deemed worth the additional effort. Increased connectivity with apathy might be a compensatory mechanism reflecting underlying neurochemical disruptions such as dopamine depletion.^43^, ^44^ This disruption affects the bottom‐up processes of approach/avoidance behavior based on reward and effort information, potentially altering drive and motivation.^45^, ^46^ Alternatively, the NA's interaction with the FPN and other cortical regions might be involved in cognitive control and working memory processes, mobilizing cognitive resources to achieve goals and guide decisions accordingly.^47^, ^48^ Such regulation assumes a top‐down functioning and might be chemically mediated by other neurotransmitters such as acetylcholine and norepinephrine.^9^, ^17^ These mechanisms might be more related to cognitive apathy, with a growing body of literature highlighting the role of these neurotransmitters and pathways in the syndrome.^9^ Understanding the specific mechanisms through which the NA influences EBDM may provide insights into potential therapeutic targets for addressing motivational deficits in neurodegenerative diseases and other conditions. However, it is crucial to note that the functional connectivity of these connections is not exclusive to EBDM. Their involvement in apathy and motivation should be considered within the broader spectrum of their functions. The NA, for instance, also plays a significant role in emotional regulation and social behavior.^47^ Its interactions with the hippocampus and amygdala play a role in processing contextual and emotional aspects of experiences, which can influence motivational states and decision‐making processes.^47^, ^49^ The integration of these signals helps in forming adaptive responses to environmental stimuli, balancing the need for reward against potential risks or punishments.^47^ This is in addition to cortical connectivity which facilitates complex cognitive functions, including attention and working memory.^50^

This study has limitations. Variations in sample sizes affected some analyses (eg, lower number of AMI‐CG reports and MRI scans), and the absence of significant correlations between EBDM measures and functional connectivity limits mechanistic interpretation. Further research with larger samples is needed. Despite these limitations, the study highlights shared EBDM deficits in AD and SCI, correlated with apathy and executive dysfunction, and associated with striatal‐cortical connectivity.

## AUTHOR CONTRIBUTIONS

Bahaaeddin Attaallah and Masud Husain designed the study. Bahaaeddin Attaallah, Sofia Toniolo, and Maria Raquel Maio collected the data. Bahaaeddin Attaallah analyzed the data and wrote the paper with input from Masud Husain. All authors reviewed the paper.

## CONFLICT OF INTEREST STATEMENT

The authors declare no competing interests. Author disclosures are available in the Supporting Information.

## CONSENT STATEMENT

All participants provided informed consent.

## Supporting information



Supporting Information

Supporting Information
