# Supplementary material for: Apathy and effort‐based decision‐making in Alzheimer's disease and subjective cognitive impairment
Source: Alzheimers Dement (Amst). 2024 Oct 16;16(4):e70013. doi: 10.1002/dad2.70013 (PMC11480904; doi:10.1002/dad2.70013)
Supplement: Supplementary file 1 — Supporting Information [file DAD2-16-e70013-s002.pdf]

Supplementary information

Additional Methods

Clinical evaluation of patients AD and SCI participants were clinically assessed by trained neurologists (co-authors M.H. & S.T.) in the cognitive disorders clinic at John Radcliff Hospital, Oxford. In addition to detailed clinical assessment, the diagnostic process included standardised objective cognitive assessment using Addenbrooke's Cognitive Examination (ACE-III), MRI imaging, as well as FDG PET when clinically indicated. AD patients had evidence of cognitive impairment along with clinical investigations consistent with AD pathology, and diagnosis was based on clinical and imaging criteria without necessarily obtaining biomarkers. SCI patients by definition had normal objective cognitive performance and brain imaging.[1]

Running EBDM task At the beginning of the study, an experienced researcher explained the task to the participants using visual illustrations and examples. Then, MVC was obtained by asking the participant to squeeze as hard as possible for three attempts; the highest of the three recordings was subsequently used as the MVC reference for the effort levels in the task. Following this, participants had the chance to practice a few decisions before moving on to respond to the 125 decisions of the experiments. Responses were recorded by pressing either the left or the right arrow on the keyboard pointing to the location of preferred option (Yes or No) on the screen. Instead of requiring participants to squeeze and exert effort for each offer they accepted, a total of 10 trials were randomly selected at the end of the experiments for the participants to play if they had answered yes when responding to the offers. They were told that they would receive monetary rewards based on the total number of apples they managed to collect. In practice, participants were compensated at a rate of £10 per hour.

Behavioural testing took place in a purpose-built quiet room with only the researcher and participant present. A 17-inch touchscreen PC using MATLAB version 2018 (MathWorks; https://uk.mathworks.com) and Psychtoolbox80 (version 3) was used to run the task. Effort responses were obtained using a hand-held dynamometer (SS25LA, BIOPAC Systems) recording from the dominant hand.

Details of statistical analyses To evaluate differences between cohorts across demographic variables, a combination of statistical tests was employed based on the data type and distribution. Continuous variables were tested for normality using the Shapiro-Wilk test and for homogeneity of variances using Levene's test. Depending on the results, either one-way ANOVA followed by Tukey-Kramer post-hoc tests (for normally distributed data with equal variances), Welch's ANOVA followed by Games-Howell post-hoc tests (for normally distributed data with unequal variances), or the Kruskal-Wallis test followed by Dunn-Sidak post-hoc tests (for non-normal data) was applied. Categorical variables (e.g., gender) were analyzed using the Chi-square test of independence.

The initial analyses of raw behavioural data (choices and decision times) were performed using mixed-effect models. These models were fitted using $fitglm$ in MATLAB v2021a, controlling for age and gender. For each of these dependent variables (choice and decision times), the models examined the effects of reward, effort and group along with their interaction. Controlling for age and gender was achieved by adding these two variables and their interaction with reward and effort as independent variables as well. Random effects of reward, effort and their interaction per participants were also included in the models. A quadratic expression of effort was used similar to previous comparable studies.[2] Decision times were log-transformed to achieve normality, and trimmed with exclusion of outliers that were three standard deviations above or below the group mean. Full description of mixed effects models and their statistical results are reported in Tables S1 & S2).

Within group robust linear regression models using *fitlm* functions in MATLAB v2021a were used to investigate the correlation between effort sensitivity (extracted from DDM, see below) as the dependent variable and measures of apathy (AMI or AMI-CG), depression (BDI-II), executive function (DS Backward), global cognition (ACE-III), age and gender as independent variables. Similar exploratory robust linear regression models were subsequently used to investigate the relationship with other DDM task parameters. Family-wise error rate correction was performed here for six multiple comparisons within each group using the Bonferroni method.

The relationship between functional connectivity (three groups of connections) and apathy (AMI) as well as effort sensitivity were also investigated within groups using similar robust linear regression models with the same covariates. These were also corrected for multiple comparisons (six within each group) using Benferroni method. All frequentist statistical tests were

two-tailed with a testing level (alpha) of 0.05.

Drift diffusion model To gain a deeper understanding of behavioural differences and the underlying psychological processes in decision-making, we combined analysis of choices and decision times using the Hierarchical Drift Diffusion Model (DDM). This computational model has been previously validated in studies on small vessel disease[3] and schizophrenia[4]. The DDM conceptualises decision-making as a process of noisy evidence accumulation towards either acceptance or rejection of an offer.

The rate of evidence accumulation, termed the drift rate (v), was modelled as a linear function of reward (r) and squared effort (e) as follows:

$$v_{r,e} = v_0 + v_r - v_e - v_{r*e} \tag{2}$$

This model yielded seven key parameters: three parameters capturing the effects of reward (v_r), effort (v_e), and their interaction ($v_{r\times e}$) on the drift rate; and four basic parameters including the baseline drift rate (v_0), the decision threshold (a) which represents the distance between the decision boundaries (accept and reject), the initial bias (z) reflecting the starting point of evidence accumulation, and the non-decision time (t) which accounts for processes such as motor preparation and perceptual encoding. Due to the task design, the parameters a, z, and t were held constant across all conditions.

Data fitting was performed using the Hierarchical Drift Diffusion Model (HDDM) toolbox (`http://ski.clps.brown.edu/hddm_docs/`; version 0.6.0) within a Jupyter notebook environment running Python 3. Bayesian significance testing was employed to assess the results, with posterior probabilities greater than 0.95 considered indicative of significant effects.

Model convergence was rigorously evaluated using Gelman-Rubin statistics (R-hat) across five Markov Chain Monte Carlo (MCMC) chains for the three study groups. All R-hat values were below 1.1, confirming satisfactory convergence for all model parameters (Table S3). The sampling procedure involved generating 20,000 samples, with the first 5,000 samples designated as burn-in and thus excluded from the final analysis to ensure stability and accuracy of the parameter estimates.

fMRI CONN processing pipeline The pipeline performs functional realignment and unwarping, slice-timing and motion correction, segmentation, and normalisation to MNI space. Spatial smoothing was applied in using spatial convolution with Gaussian kernel of 8 mm full width half maximum. Following this, linear regression controlling for potential confounds and with temporal band-pass filtering (0.008 - 0.09 Hz) was applied for denoising. The model controlled for noise signals originating from white matter, cerebrospinal fluid, head motion, and confounding effects arising from identified outliers and from linear BOLD signal trends.

Additional Results

Demographics AD patients were generally older than HC and SCI participants (Age; HC: $\mu = 62.11,\ SD = 10.04$, AD: $\mu = 68.67,\ SD = 9.91$, SCI: $\mu = 58.17,\ SD = 7.82$, $p < 0.001$; Table 1), and there was no difference in male to female ratio across the three groups (p = 0.67).

Cognitive scores indexed by ACE-III were significantly different across the three groups (ACE-III; HC: $\mu = 97.35,\ SD = 2.41$, AD: $\mu = 78.20,\ SD = 11.18$, SCI: $\mu = 95.00,\ SD = 3.94$, $p < 0.001$), with AD having the lowest scores, followed by SCI and then HC. All SCI participants had ACE-III scores above 88 consistent with the group characterisation of having normal objectively measured cognitive in SCI.

AD patients also showed lower total Digit Span (DS) scores compared to both HC and SCI groups (DS; HC: $\mu = 17.81,\ SD = 2.72$, AD: $\mu = 15.47,\ SD = 4.28$, SCI: $\mu = 18.83,\ SD = 4.16$, $p < 0.01$). Total DS scores were not significantly different between SCI and HC (DS; HC: $\mu = 17.81,\ SD = 2.72$, SCI: $\mu = 18.83,\ SD = 4.16$, $p = 0.91$). Examining DS domains demonstrated that AD patients had lower backward DS compared to HC, and preserved Forward component, indicating a deficit in executive function (DS Backward; HC: $\mu = 7.36,\ SD = 1.63$, AD: $\mu = 5.77,\ SD = 2.50$, $p < 0.01$) and less impairment in forward DS (HC: $\mu = 10.45,\ SD = 1.91$, AD: $\mu = 9.70,\ SD = 2.23$, $p = 0.29$).

Additional discussion

The AD sample in this study did not report higher self-apathy scores compared to the other groups. However, caregiver reports suggested that patients were significantly more apathetic

than self-reported and compared to HC. SCI participants, on the other hand, reported significantly higher levels of apathy, which were similar to their informant estimations. On the EBDM task, both AD and SCI participants, compared to HC, demonstrated reduced sensitivity to effort as indexed by the influence of effort on drift rate in the DDM, with AD showing more severe blunting overall. This effect correlated with apathy scores in SCI and executive dysfunction in AD. Further, the three groups differed in their functional connectivity patterns across different networks including a connection involving the Nucleus Accumbens (NA) (ventral striatum) and Posterior Parietal Cortex (PPC) region in Fronto-Parietal Network (FPN). The strength of this connection correlated with the severity of apathy in SCI. These results suggest a partially shared decision-making and functional connectivity profile in AD and SCI that might have different implications across the AD spectrum on motivation and executive functioning.

In the context of EBDM, behavioural apathy has been traditionally linked to at least two key mechanisms: increased aversiveness of effort or/and decreased incentivisation by rewards.[5–8] It should be noted that the term '*sensitivity*' in our study describes how steep the slope of offer acceptance or drift rate is as a function of changes in effort (or reward), rather than describing absolute effects. In other words, it describes to what extent participants take changes in effort (or reward) level into consideration when they compute subjective values and assign preferences. AD and SCI participants were considered less sensitive to effort primarily according to this characterisation, without necessarily reflecting aversiveness of certain effort levels. However, preference patterns suggest that AD and SCI participants were less willing to accept low-effort offers, indicating subjectively higher discounting effect of low effort in these groups — a pattern that is behaviourally apathetic.

Examining decision-making in patients with cognitive dysfunction can be challenging as this can distort the interpretation of results and participants' behaviour. A fundamental question is 'Are we measuring what we think we are measuring?'.[9–11] While every effort has been made to ensure that this is the case in this study, e.g., making sure participants understood the task using post-instruction questionnaires and excluding AD patients with doubts regarding their understanding of the paradigm, it remains necessary to consider this question before jumping to conclusions about AD patients' behaviour.

Here, we consider two parallel lines of interpretations of the main findings. First, reduced sensitivity to effort in AD might indeed be indicative of lower weights assigned to changes

in effort when making value-based decisions, reflecting reduced motivational drives (i.e., behavioural apathy). This decision-making profile is similar but more severe than the one observed in apathetic SCI individuals, especially when modelling decision-making using the DDM. Such deficits have shown AD patients making decisions that were more weighed by their reward attributes, resulting in seemingly irrational and less profitable preferences (e.g., low-reward, high-effort), a finding that has been partially highlighted in one previous behavioural investigation in AD using the Iowa Gambling Task.[12] However, unlike in SCI, where blunted effort sensitivity reflected their apathy, these deficits in effort-based decision-making (EBDM) were not related to apathy scores in AD but alternatively to another marker of disease severity, executive dysfunction.

This opens the possibility that AD patients might be less able to compute subjective values that involve more than one attribute simultaneously. This might reflect inability to allocate cognitive resources properly to guide decision-making processes. A mechanism that has been proposed for a different type of apathy – cognitive apathy.[13–15] The fact that effort sensitivity correlates with executive function supports this conclusion. Importantly, this does not seem to be related to global cognitive dysfunction (no correlation between V_e and ACE-III scores), pointing to a more specific dysexecutive component of EBDM value computations in AD. The distinction between global cognitive decline and executive deficits has also been reported in previous studies in different domains such as decision-making under risk and ambiguity (for a recent review see REF[16]). However, if this is indeed the case, then why does it affect the effort component more than reward? Does this reflect a genuine prioritisation of reward information over effort when agents have limited cognitive resources? Or is this related to task-specific factors such as more salient reward cues compared to effort ones? These questions could be answered in follow-up studies aiming to investigate how AD patients attend to, interpret and integrate reward and effort information, e.g., by manipulating how effort and reward are presented and delivered, with a more detailed examination of executive function deficits in this group.

Another challenge with examining the correlation between task measures and apathy scores is to ascertain how reliable self-reports of apathy scores are. Lack of insight is a prominent feature in AD,[17,18] which might affect their motivation rating. We have attempted to overcome this by using scores from informants. Despite the fact that AMI-CG scores mapped well onto self-reported scores in AD and revealed that patients might underestimate their motivation, no major

differences were noted with regard to correlations with effort-based decision-making (EBDM) parameters from the DDM. While decision bias showed a significant correlation with caregiver apathy scores, this result was from an exploratory analysis that was likely underpowered and did not survive multiple correlations corrections. However, this preliminary observation suggests that there might be a degree of dissociation between different sources of apathy reports in AD patients and that they might map to different decision-making attributes. For example, it might highlight a potential mechanism of apathy in AD that involves alterations of internal motivation when evaluating effort against reward, with apathetic patients having less initial bias when making decisions. Such a 'neutral' state when approaching decisions resonates with the classical description of apathy as simply being 'not bothered'.[19–21] It also accords with recent investigations suggesting that apathy is related to decision inertia, whereby more apathetic people tend to take on more decisions despite the effort involved.[22] It would be worth attempting to validate such a finding in a larger group and in other forms of decision-making (e.g., risk-based decisions, decision-making under uncertainty).

Supplementary Tables

Tables S1 to S3

	Effort-Based Decisions	Decision Time
(Intercept)	$\beta = +1.89$<br>$SE = 0.22$<br>$t_{15721} = +8.60$<br>***p<0.0001***	$\beta = +0.788$<br>$SE = 0.0322$<br>$t_{15650} = +24.50$<br>***p<0.0001***
AD	$\beta = -0.813$<br>$SE = 0.374$<br>$t_{15721} = -2.17$<br>***p = 0.03***	$\beta = +0.329$<br>$SE = 0.0562$<br>$t_{15650} = +5.86$<br>***p<0.0001***
AD:Effort	$\beta = +1.22$<br>$SE = 0.282$<br>$t_{15721} = +4.31$<br>***p<0.0001***	$\beta = -0.0254$<br>$SE = 0.0124$<br>$t_{15650} = -2.04$<br>***p = 0.041***
AD:Reward	$\beta = -0.644$<br>$SE = 0.341$<br>$t_{15721} = -1.89$<br>$p = 0.06$	$\beta = +0.00528$<br>$SE = 0.0127$<br>$t_{15650} = +0.42$<br>$p = 0.68$
AD:Reward:Effort	$\beta = -0.635$<br>$SE = 0.267$<br>$t_{15721} = -2.38$<br>***p = 0.018***	$\beta = -0.0261$<br>$SE = 0.00952$<br>$t_{15650} = -2.74$<br>***p = 0.0061***
SCI	$\beta = -0.171$<br>$SE = 0.34$<br>$t_{15721} = -0.50$<br>$p = 0.61$	$\beta = +0.126$<br>$SE = 0.05$<br>$t_{15650} = +2.51$<br>***p = 0.012***
SCI:Effort	$\beta = +0.414$<br>$SE = 0.262$<br>$t_{15721} = +1.58$<br>$p = 0.11$	$\beta = -0.0112$<br>$SE = 0.011$<br>$t_{15650} = -1.01$<br>$p = 0.31$
SCI:Reward	$\beta = +0.463$<br>$SE = 0.316$<br>$t_{15721} = +1.47$<br>$p = 0.14$	$\beta = -0.00823$<br>$SE = 0.0113$<br>$t_{15650} = -0.73$<br>$p = 0.47$
SCI:Reward:Effort	$\beta = -0.299$<br>$SE = 0.25$<br>$t_{15721} = -1.19$<br>$p = 0.23$	$\beta = -0.0111$<br>$SE = 0.00845$<br>$t_{15650} = -1.32$<br>$p = 0.19$
Age	$\beta = +0.148$<br>$SE = 0.155$<br>$t_{15721} = +0.95$<br>$p = 0.34$	$\beta = +0.0653$<br>$SE = 0.023$<br>$t_{15650} = +2.84$<br>***p = 0.0046***
Age:Effort	$\beta = +0.069$<br>$SE = 0.118$<br>$t_{15721} = +0.58$<br>$p = 0.56$	$\beta = +0.00348$<br>$SE = 0.00509$<br>$t_{15650} = +0.68$<br>$p = 0.49$
Age:Reward	$\beta = -0.00677$<br>$SE = 0.144$<br>$t_{15721} = -0.05$<br>$p = 0.96$	$\beta = +0.00919$<br>$SE = 0.0052$<br>$t_{15650} = +1.77$<br>$p = 0.08$
Age:Reward:Effort	$\beta = -0.206$<br>$SE = 0.113$<br>$t_{15721} = -1.82$<br>$p = 0.07$	$\beta = -0.00773$<br>$SE = 0.0039$<br>$t_{15650} = -1.98$<br>***p = 0.047***
Gender	$\beta = +0.115$<br>$SE = 0.143$<br>$t_{15721} = +0.80$<br>$p = 0.42$	$\beta = +0.0583$<br>$SE = 0.0213$<br>$t_{15650} = +2.74$<br>***p = 0.0062***
Gender:Effort	$\beta = +0.196$<br>$SE = 0.109$<br>$t_{15721} = +1.79$<br>$p = 0.07$	$\beta = +0.00612$<br>$SE = 0.0047$<br>$t_{15650} = +1.30$<br>$p = 0.19$
Gender:Reward	$\beta = +0.25$<br>$SE = 0.132$<br>$t_{15721} = +1.89$<br>$p = 0.06$	$\beta = -0.00183$<br>$SE = 0.00481$<br>$t_{15650} = -0.38$<br>$p = 0.70$
Gender:Reward:Effort	$\beta = -0.0705$<br>$SE = 0.104$<br>$t_{15721} = -0.68$<br>$p = 0.50$	$\beta = -0.00283$<br>$SE = 0.0036$<br>$t_{15650} = -0.79$<br>$p = 0.43$
Effort	$\beta = -2.35$<br>$SE = 0.169$<br>$t_{15721} = -13.91$<br>***p<0.0001***	$\beta = +0.0458$<br>$SE = 0.00709$<br>$t_{15650} = +6.46$<br>***p<0.0001***
Reward	$\beta = +3.04$<br>$SE = 0.202$<br>$t_{15721} = +15.09$<br>***p<0.0001***	$\beta = -0.0218$<br>$SE = 0.00726$<br>$t_{15650} = -3.01$<br>***p = 0.0026***
Reward:Effort	$\beta = +0.475$<br>$SE = 0.159$<br>$t_{15721} = +2.99$<br>***p = 0.0028***	$\beta = +0.0565$<br>$SE = 0.00543$<br>$t_{15650} = +10.40$<br>***p<0.0001***
$adj - R^2$	1.00	0.44
N_{obs}	15741	15670
AIC	9173.98	11159.07

Table S1: **Generalised mixed effect models investigating group differences in choices and decision time with HC as baseline group.** Models were specified as follows: Predicted variable (choice or logDT) $\sim$ 1 + group*Reward + Age*Reward + Gender*Reward + group*Effort + Age*Effort + Gender*Effort + Reward*Effort + group:Reward:Effort + Age:Reward:Effort + Gender:Reward:Effort + (1 + Reward*Effort |participant).

	Effort-Based Decisions	Decision Time
(Intercept)	$\beta = +1.72$<br>$SE = 0.261$<br>$t_{15721} = +6.57$<br>***p<0.0001***	$\beta = +0.913$<br>$SE = 0.0384$<br>$t_{15650} = +23.77$<br>***p<0.0001***
HC	$\beta = +0.171$<br>$SE = 0.34$<br>$t_{15721} = +0.50$<br>$p = 0.61$	$\beta = -0.126$<br>$SE = 0.05$<br>$t_{15650} = -2.51$<br>***p = 0.012***
HC:Effort	$\beta = -0.414$<br>$SE = 0.262$<br>$t_{15721} = -1.58$<br>$p = 0.11$	$\beta = +0.0112$<br>$SE = 0.011$<br>$t_{15650} = +1.01$<br>$p = 0.31$
HC:Reward	$\beta = -0.463$<br>$SE = 0.316$<br>$t_{15721} = -1.47$<br>$p = 0.14$	$\beta = +0.00823$<br>$SE = 0.0113$<br>$t_{15650} = +0.73$<br>$p = 0.47$
HC:Reward:Effort	$\beta = +0.299$<br>$SE = 0.25$<br>$t_{15721} = +1.19$<br>$p = 0.23$	$\beta = +0.0111$<br>$SE = 0.00845$<br>$t_{15650} = +1.32$<br>$p = 0.19$
AD	$\beta = -0.642$<br>$SE = 0.414$<br>$t_{15721} = -1.55$<br>$p = 0.12$	$\beta = +0.204$<br>$SE = 0.0621$<br>$t_{15650} = +3.28$<br>***p = 0.001***
AD:Effort	$\beta = +0.801$<br>$SE = 0.313$<br>$t_{15721} = +2.56$<br>***p = 0.011***	$\beta = -0.0142$<br>$SE = 0.0137$<br>$t_{15650} = -1.04$<br>$p = 0.30$
AD:Reward	$\beta = -1.11$<br>$SE = 0.38$<br>$t_{15721} = -2.91$<br>***p = 0.0036***	$\beta = +0.0135$<br>$SE = 0.014$<br>$t_{15650} = +0.96$<br>$p = 0.34$
AD:Reward:Effort	$\beta = -0.336$<br>$SE = 0.3$<br>$t_{15721} = -1.12$<br>$p = 0.26$	$\beta = -0.015$<br>$SE = 0.0105$<br>$t_{15650} = -1.43$<br>$p = 0.15$
Age	$\beta = +0.148$<br>$SE = 0.155$<br>$t_{15721} = +0.95$<br>$p = 0.34$	$\beta = +0.0653$<br>$SE = 0.023$<br>$t_{15650} = +2.84$<br>***p = 0.0046***
Age:Effort	$\beta = +0.069$<br>$SE = 0.118$<br>$t_{15721} = +0.58$<br>$p = 0.56$	$\beta = +0.00348$<br>$SE = 0.00509$<br>$t_{15650} = +0.68$<br>$p = 0.49$
Age:Reward	$\beta = -0.00677$<br>$SE = 0.144$<br>$t_{15721} = -0.05$<br>$p = 0.96$	$\beta = +0.00919$<br>$SE = 0.0052$<br>$t_{15650} = +1.77$<br>$p = 0.08$
Age:Reward:Effort	$\beta = -0.206$<br>$SE = 0.113$<br>$t_{15721} = -1.82$<br>$p = 0.07$	$\beta = -0.00773$<br>$SE = 0.0039$<br>$t_{15650} = -1.98$<br>***p = 0.047***
Gender	$\beta = +0.115$<br>$SE = 0.143$<br>$t_{15721} = +0.80$<br>$p = 0.42$	$\beta = +0.0583$<br>$SE = 0.0213$<br>$t_{15650} = +2.74$<br>***p = 0.0062***
Gender:Effort	$\beta = +0.196$<br>$SE = 0.109$<br>$t_{15721} = +1.79$<br>$p = 0.07$	$\beta = +0.00612$<br>$SE = 0.0047$<br>$t_{15650} = +1.30$<br>$p = 0.19$
Gender:Reward	$\beta = +0.25$<br>$SE = 0.132$<br>$t_{15721} = +1.89$<br>$p = 0.06$	$\beta = -0.00183$<br>$SE = 0.00481$<br>$t_{15650} = -0.38$<br>$p = 0.70$
Gender:Reward:Effort	$\beta = -0.0705$<br>$SE = 0.104$<br>$t_{15721} = -0.68$<br>$p = 0.50$	$\beta = -0.00283$<br>$SE = 0.0036$<br>$t_{15650} = -0.79$<br>$p = 0.43$
Effort	$\beta = -1.93$<br>$SE = 0.2$<br>$t_{15721} = -9.65$<br>***p<0.0001***	$\beta = +0.0347$<br>$SE = 0.00847$<br>$t_{15650} = +4.09$<br>***p<0.0001***
Reward	$\beta = +3.51$<br>$SE = 0.244$<br>$t_{15721} = +14.37$<br>***p<0.0001***	$\beta = -0.03$<br>$SE = 0.00867$<br>$t_{15650} = -3.47$<br>***p = 0.00053***
Reward:Effort	$\beta = +0.176$<br>$SE = 0.194$<br>$t_{15721} = +0.91$<br>$p = 0.36$	$\beta = +0.0454$<br>$SE = 0.00649$<br>$t_{15650} = +6.99$<br>***p<0.0001***
$adj - R^2$	1.00	0.44
N_{obs}	15741	15670
AIC	9173.98	11159.07

Table S2: **Generalised mixed effect models investigating group differences in choices and decision time with SCI as baseline group.** Models were specified as follows: Predicted variable (choice or logDT) $\sim$ 1 + group*Reward + Age*Reward + Gender*Reward + group*Effort + Age*Effort + Gender*Effort + Reward*Effort + group:Reward:Effort + Age:Reward:Effort + Gender:Reward:Effort + (1 + Reward*Effort |participant). HC group was used as reference.

Parameter	HC	AD	SCI
Threshold (a)	0.999957	0.999972	1.000065
Non-Decision Time (t)	1.000279	1.000016	0.999981
Decision Bias (z)	1.000062	1.004861	1.000258
Intercept	0.999992	1.000607	0.999975
V:Effort	1.000164	0.999996	1.000008
V:Reward	0.999952	1.000022	0.999972
V:Effort:Reward	1.000066	1.000160	0.999990

Table S3: Gelman Rubin statistics (R-hat) across five MCMC chains for the three study groups. All R-hat values are less than 1.1 indicating Model convergence for all parameters. Model sampling included 20000 sample, 5000 of which were burn samples.

References

[1] F. Jessen, R. E. Amariglio, R. F. Buckley, W. M. van der Flier, Y. Han, J. L. Molinuevo, L. Rabin, D. M. Rentz, O. Rodriguez-Gomez, A. J. Saykin, S. A. M. Sikkes, C. M. Smart, S. Wolfsgruber, and M. Wagner, "The characterisation of subjective cognitive decline," *The Lancet Neurology*, vol. 19, pp. 271–278, 3 2020.

[2] T. T.-J. Chong, M. Apps, K. Giehl, A. Sillence, L. L. Grima, and M. Husain, "Neurocomputational mechanisms underlying subjective valuation of effort costs," *PLOS Biology*, vol. 15, p. e1002598, 2 2017.

[3] Y. Saleh, C. Le Heron, P. Petitet, M. Veldsman, D. Drew, O. Plant, U. Schulz, A. Sen, P. M. Rothwell, S. Manohar, and M. Husain, "Apathy in small vessel cerebrovascular disease is associated with deficits in effort-based decision making," *Brain*, vol. 144, pp. 1247–1262, 5 2021.

[4] Y. Saleh, I. Jarratt-Barnham, P. Petitet, E. Fernandez-Egea, S. G. Manohar, and M. Husain, "Negative symptoms and cognitive impairment are associated with distinct motivational deficits in treatment resistant schizophrenia," *Molecular Psychiatry 2023*, pp. 1–11, 8 2023.

[5] N. Sinha, S. Manohar, and M. Husain, "Impulsivity and apathy in Parkinson's disease," *Journal of Neuropsychology*, vol. 7, pp. 255–283, 9 2013.

[6] M. Husain and J. P. Roiser, "Neuroscience of apathy and anhedonia: a transdiagnostic approach," *Nature Reviews Neuroscience*, vol. 19, pp. 470–484, 8 2018.

[7] T. T. Chong and M. Husain, "The role of dopamine in the pathophysiology and treatment of apathy," *Progress in Brain Research*, vol. 229, pp. 389–426, 1 2016.

[8] Y. Saleh, I. Jarratt-Barnham, E. Fernandez-Egea, and M. Husain, "Mechanisms Underlying Motivational Dysfunction in Schizophrenia," *Frontiers in Behavioral Neuroscience*, vol. 15, 9 2021.

[9] I.-C. A. Chiang, R. S. Jhangiani, and P. C. Price, "Reliability and Validity of Measurement," 10 2015.

[10] J. K. Flake and E. I. Fried, "Measurement Schmeasurement: Questionable Measurement Practices and How to Avoid Them," *https://doi.org/10.1177/2515245920952393*, vol. 3, pp. 456–465, 12 2020.

[11] J. Snodgrass, G. Levy-Berger, and M. Haydon, *Human experimental psychology*. 1985.

[12] S. Bayard, J. P. Jacus, S. Raffard, and M. C. Gely-Nargeot, "Apathy and emotion-based decision-making in amnesic mild cognitive impairment and Alzheimer's disease," *Behavioural Neurology*, vol. 2014, 2014.

[13] R. Levy and B. Dubois, "Apathy and the Functional Anatomy of the Prefrontal Cortex–Basal Ganglia Circuits," *Cerebral Cortex*, vol. 16, pp. 916–928, 7 2006.

[14] J. Pagonabarraga, J. Kulisevsky, A. P. Strafella, and P. Krack, "Apathy in Parkinson's disease: clinical features, neural substrates, diagnosis, and treatment," *The Lancet Neurology*, vol. 14, pp. 518–531, 5 2015.

[15] M. Béreau, V. Van Waes, M. Servant, E. Magnin, L. Tatu, and M. Anheim, "Apathy in Parkinson's Disease: Clinical Patterns and Neurobiological Basis," *Cells 2023, Vol. 12, Page 1599*, vol. 12, p. 1599, 6 2023.

[16] F. Gaubert and H. Chainay, "Decision-Making Competence in Patients with Alzheimer's Disease: A Review of the Literature," *Neuropsychology Review 2021 31:2*, vol. 31, pp. 267–287, 2 2021.

[17] B. R. Ott, R. B. Noto, and B. S. Fogel, "Apathy and loss of insight in Alzheimer's disease: a SPECT imaging study," *https://doi.org/10.1176/jnp.8.1.41*, vol. 8, pp. 41–46, 4 2006.

[18] S. M. Horning, R. Melrose, and D. Sultzer, "Insight in Alzheimer's disease and its relation to psychiatric and behavioral disturbances," *International Journal of Geriatric Psychiatry*, vol. 29, pp. 77–84, 1 2014.

[19] W. Baber, C. Y. M. Chang, J. Yates, and T. Dening, "The Experience of Apathy in Dementia: A Qualitative Study," *International Journal of Environmental Research and Public Health 2021, Vol. 18, Page 3325*, vol. 18, p. 3325, 3 2021.

[20] J. Simpson, H. McMillan, I. Leroi, and C. D. Murray, "Experiences of apathy in people with Parkinson's disease: a qualitative exploration," *https://doi.org/10.3109/09638288.2014.939771*, vol. 37, pp. 611–619, 4 2015.

[21] B. R. Stanton, A. Carson, D. Biba, and R. Stanton, "Apathy: a practical guide for neurologists," *Practical Neurology*, vol. 16, pp. 42–47, 2 2016.

[22] J. Scholl, H. A. Trier, M. F. Rushworth, and N. Kolling, "The effect of apathy and compulsivity on planning and stopping in sequential decision-making," *PLOS Biology*, vol. 20, p. e3001566, 3 2022.
